# The Hidden Pandemic of Family Violence During COVID-19: Unsupervised Learning of Tweets

**DOI:** 10.2196/24361

**Published:** 2020-11-06

**Authors:** Jia Xue, Junxiang Chen, Chen Chen, Ran Hu, Tingshao Zhu

**Affiliations:** 1 Factor-Inwentash Faculty of Social Work University of Toronto Toronto, ON Canada; 2 Faculty of Information University of Toronto Toronto, ON Canada; 3 School of Medicine University of Pittsburgh Pittsburgh, PA United States; 4 Middleware System Research Group University of Toronto Toronto, ON Canada; 5 Institute of Psychology Chinese Academy of Sciences Beijing China

**Keywords:** Twitter, family violence, COVID-19, machine learning, big data, infodemiology, infoveillance

## Abstract

**Background:**

Family violence (including intimate partner violence/domestic violence, child abuse, and elder abuse) is a hidden pandemic happening alongside COVID-19. The rates of family violence are rising fast, and women and children are disproportionately affected and vulnerable during this time.

**Objective:**

This study aims to provide a large-scale analysis of public discourse on family violence and the COVID-19 pandemic on Twitter.

**Methods:**

We analyzed over 1 million tweets related to family violence and COVID-19 from April 12 to July 16, 2020. We used the machine learning approach Latent Dirichlet Allocation and identified salient themes, topics, and representative tweets.

**Results:**

We extracted 9 themes from 1,015,874 tweets on family violence and the COVID-19 pandemic: (1) increased vulnerability: COVID-19 and family violence (eg, rising rates, increases in hotline calls, homicide); (2) types of family violence (eg, child abuse, domestic violence, sexual abuse); (3) forms of family violence (eg, physical aggression, coercive control); (4) risk factors linked to family violence (eg, alcohol abuse, financial constraints, guns, quarantine); (5) victims of family violence (eg, the LGBTQ [lesbian, gay, bisexual, transgender, and queer or questioning] community, women, women of color, children); (6) social services for family violence (eg, hotlines, social workers, confidential services, shelters, funding); (7) law enforcement response (eg, 911 calls, police arrest, protective orders, abuse reports); (8) social movements and awareness (eg, support victims, raise awareness); and (9) domestic violence–related news (eg, Tara Reade, Melissa DeRosa).

**Conclusions:**

This study overcomes limitations in the existing scholarship where data on the consequences of COVID-19 on family violence are lacking. We contribute to understanding family violence during the pandemic by providing surveillance via tweets. This is essential for identifying potentially useful policy programs that can offer targeted support for victims and survivors as we prepare for future outbreaks.

## Introduction

As seen in the case of Ebola, epidemics increase the rates of domestic violence [1]. The World Health Organization declared COVID-19 a pandemic on March 11, 2020. To effectively control the spread of the disease, many countries have adopted rigorous measures to limit mobility, such as social distancing, stay-at-home orders (sheltering in place), closure of nonessential business, travel restrictions, and quarantine. Even though these measures are useful for infection control [2], they bring a series of negative social consequences, such as psychological stress [3-5], unemployment [6], ageism [7], and increased rates of violence against women and children [8-11]. Since these rigorous measures overlap with many of the intervention strategies for family violence [2], they are likely to increase the vulnerability of victims of family violence (including intimate partner violence [IPV]/domestic violence, elder abuse, and child abuse), by increasing exposure to an exploitative relationship, reducing options for support [10], economic stress [12], and alcohol abuse [13,14]. For example, isolation limits social contact with families and social services, and thus may facilitate family violence and prevent victims from seeking help [15-17]. During the COVID-19 quarantine, the home becomes a dangerous place for victims while individuals are living in forced close quarters [18]. In addition, mental health exacerbated by social isolation increases the likelihood of locking victims of domestic violence in an unsafe home environment and increases their vulnerability [19]. UNICEF [20] reports that school closures increased child (sexual) abuse and neglect during the Ebola epidemic. It is also important to note that child abuse and domestic violence are likely to co-occur when isolated at home [21,22]. During the COVID-19 pandemic, scholars have suggested that new forms of family violence may occur; for example, abusers may threaten to infect their family members with the virus [23].

In many countries, the reported cases of and service needs related to family violence dramatically increased since quarantine measures came into effect [18]. For example, calls to domestic violence hotlines have risen by 25%, and the number of Google searches for family violence–related help during the outbreak has been substantial [24]. According to National Domestic Violence Hotline representatives in the United States, abusers are attempting to isolate victims from resources and unleashing more violence by enforcing COVID-19 social distancing measures [25]. In the United Kingdom, calls to the Domestic Violence Helpline increase by 25% in the first week after the lockdown measures were implemented [26]. In China, domestic violence increased three times in Hubei Province during the lockdown [27]. There was a 10.2% increase in domestic violence calls in the United States during the COVID-19 pandemic [28]. These reports illustrate that existing COVID-19 intervention measures (eg, living in a closed space with abusers for a long period) may profoundly impact victims and survivors of family violence. According to Bradbury-Jones and Isham [8], “domestic violence rates are rising, and they are rising fast” (p 2047). Data on family violence during the pandemic are still scarce [29], and there is a need for further research.

We cannot capture the impact of COVID-19 on family violence without adequate surveillance [30]. Enhanced surveillance provides an understanding of the impact and risk factors associated with COVID-19, which is essential for developing policy programs to respond and mitigate adverse effects and offer targeted support for victims and survivors [30]. Eysenbach [31] defined infodemiology and infoveillance as “the science of distribution and determinants of information in an electronic medium, specifically the Internet, or in a population, with the ultimate aim to inform public health and public policy. Infodemiology data can be collected and analyzed in near real time” (p 1). According to Eysenbach’s framework, the automated analysis of unstructured data related to family violence and COVID-19 is an application of an infoveillance study. Understanding public discussions can assist governments and public health authorities in navigating the outbreak [32].

During the implementation of social isolation measures, social media should be leveraged to raise public awareness and share best practices (eg, bystander approaches, supportive statements, obtaining help on behalf of a survivor) [2], and provide support [33]. Twitter is a real-time network that allows users from across the globe to communicate via public and private messages, organized chronologically on a given user's account. Existing studies have confirmed Twitter's role in connecting practitioners and clients [34-36]. Researchers have used Twitter data to examine the nature of domestic violence [37-39]. A significant number of studies describe Twitter hashtag #MeToo as a phenomenal tool for disclosing experiences of sexual harassment, and more importantly, to ignite a widespread social campaign or political protest on social media. Modrek and Chakalov [40] examined tweets containing #MeToo in the United States and supported the role of machine learning methods in understanding the widespread sexual assault self-revelations on Twitter. Recently, Twitter has become a valuable source for understanding user-generated COVID-19 content and activities in real time [41,42].

### Aim of the Study

There is a lack of data on the COVID-19 pandemic as it relates to family violence [43]. This study aims to provide a large-scale analysis of public discourse on family violence and COVID-19 on Twitter using machine learning techniques to fill this gap. The research questions are as follows: (1) what contents are discussed relating to family violence and COVID-19? and (2) what themes are identified relating to family violence and COVID-19? The study offers a new perspective on the impact, risks factors, and continuing social support services during the pandemic for family violence.

## Methods

This study employed an observational design and followed the pipeline developed by the authors [44], including sampling, data collection, preprocessing of raw data, and data analysis.

### Sampling and Data Collection

**Figure 1:**
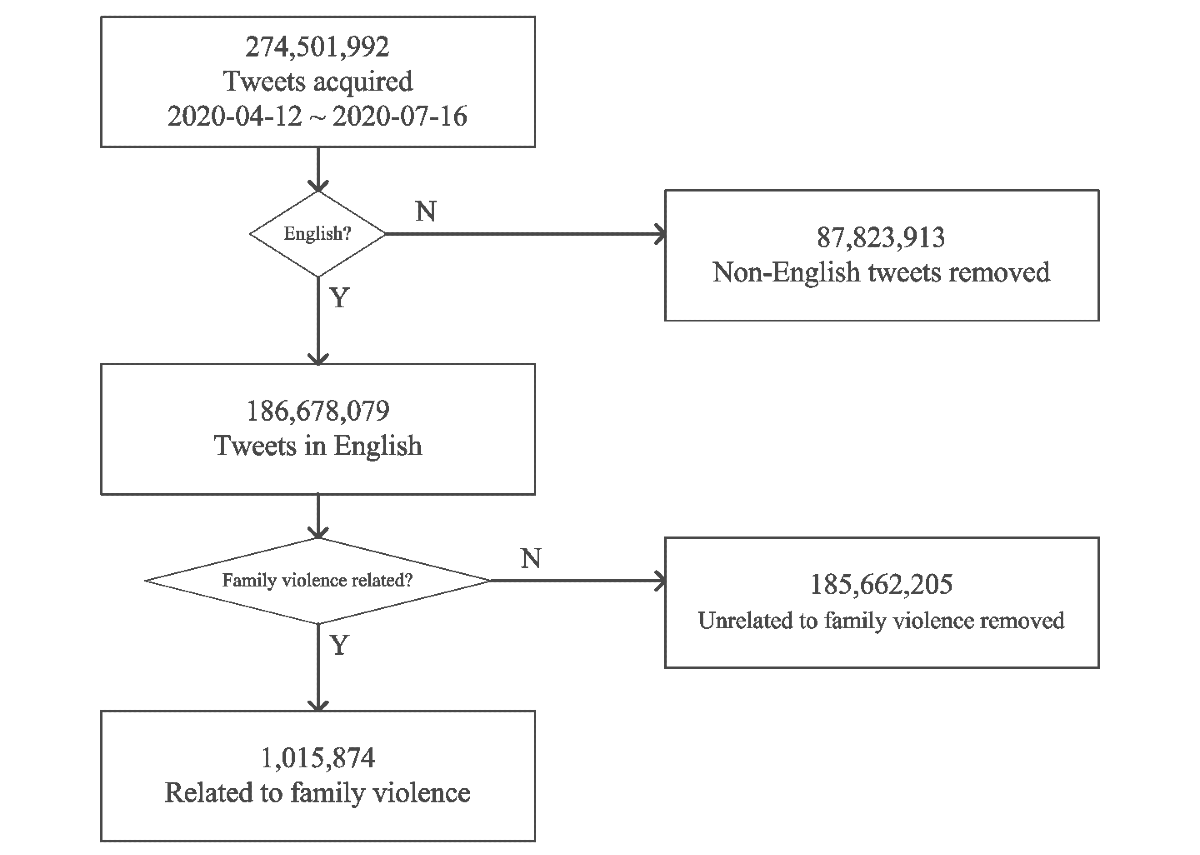
Tweets preprocessing chart.

Our COVID-19 data set used a list of COVID-19–relevant hashtags as search terms to randomly collect tweets from Twitter between April 12 to July 16, 2020 [44] (Multimedia Appendix 1). Twitter Developer’s Python code was used to access the Twitter API to collect tweets. As shown in Figure 1, our data set included a total of 274,501,992 tweets during the study period, of which 186,678,079 were in English. We sampled tweets using keywords such as “domestic violence,” “intimate partner violence,” “family violence,” “violence against women,” “gender-based violence,” “child abuse,” “child maltreatment,” “elder abuse,” and “IPV.” The final data set comprised 1,015,874 tweets.

### Preprocessing the Raw Data

We used Python to clean the data and remove the following items because they did not contribute to the semantic meaning of the tweets: the hashtag symbol, URLs, @users, special characters, punctuations, and stop-words [38,39,44,45].

### Unsupervised Machine Learning

We used a machine learning approach, Latent Dirichlet Allocation (LDA) [46], to analyze a corpus of unstructured text. LDA was a generative statistical model that regards a corpus of text (tweets) as a mixture of a small number of latent topics. Each latent topic was assigned with a set of linguistic units (eg, single words or a pair of words) counted by the algorithm. These linguistic units with high frequency were likely to co-occur and form into different latent topics. With the LDA model, the distribution of topics in documents can be inferred. LDA assumes a generative process describing how the documents are created, such that we can infer or reverse engineer the topic distributions. The generative process of LDA for M documents, each of which has a length of N_i_, is given as:

1. Choose θ_*i*_ ∼ Dir (α), with *i* ∈ {1,…,*M*}.
2. Choose *ϕ*_*k*_ ∼ Dir (*β*), with *k* ∈ {1,…,*K*}.
3. For the *j*-th linguistic unit in the *i*-th document with *i* ∈ {1,…,*M*, and*j* ∈ {1,…,*N*N_*i*_}
  - Choose *z*_*i,j*_ ∼ Multinomial (θ_*i*_)
  - Choose w*_i,j_* ∼ Multinomial (ϕ_z_i,j__)

Multimedia Appendix 2 presents the definitions of these notations. With the generative process described above, the distributions of the topics can be inferred using the Python package *genism*.

## Results

We analyzed 1,015,874 tweets mentioning family violence and COVID-19 in Twitter posts. We identified 50 latent topics and frequently mentioned pairs of words (bigrams) for each topic. We further categorized these 50 identified common topics into 9 themes and 33 topics (Table 1). Table 1 presents commonly co-occurring bigrams and examples of representative tweets under each identified theme and topic.

**Table 1:** Themes, topics, commonly co-occurring terms, and examples of tweets about domestic violence and COVID-19.

Themes and topics			Terms		Tweet example
**Increased vulnerability: COVID-19 and family violence**					
	Rising rates	violence increase, violence spike, violence risen, abuse up		“…seeking shelter at home, rates of domestic violence and abuse have skyrocketed. Further, women and girls at a high risk for trafficking…”	
	DV^a^ reports	violence reports, reports surge		“Several countries saw spikes in domestic violence reports.”	
	Hotline calls increased	crisis line, violence hotline, abuse hotline, calls increased, calls help		“…works with domestic violence survivors is seeing a spike in emergency shelter capacity and crisis line calls during the coronavirus pandemic.”	
	Homicide	murder domestic, violence homicide, murder wife		“Lots of social behaviours and issues are sadly coming to the fore during this pandemic...domestic violence where in the UK 2 women are killed...”	
	Suicide	suicide increased, suicide domestic, abuse suicide		“NY Gov. Cuomo: Suicides and increased domestic violence worth prices of coronavirus lockdown.”	
	(Mental) health	mental health, mental abuse, mental illness, abuse depression		“Longer shutdowns = even more domestic violence, more substance abuse, more lonliness. More mental health symptoms.”	
**Types of family violence during COVID-19**					
	Child abuse/maltreatment	assault child, children suffering, rape child, FGM^b^ child		“If we're going to talk about quarantine, please don't forget that children are also in danger.”	
	Domestic violence	abusive partners, risk family, family impacted		“With #StayHome orders, many women are left in isolation with abusive partners, unable to access life-saving resources and support systems.”	
	Sexual violence	sexual assault, abuse rape, marital rape, rape incest, sexual abuse		“Thousands across the country are infected … shelters for rough sleepers, sexual abused have no place to go!”	
**Forms of family violence during COVID-19**					
	Physical aggression	stop hitting, physical domestic, physical abuse, physical violence		“…violence against women and girls has risen dramatically. My fear is more women and girls will die from physical violence than #Covid19.”	
	Coercive control	power control, forced stay, coercive control, run away		“Domestic violence is about power and control. Abusers use more coercive control tactics surrounding the #covid19 pandemic to continue to maintain power and control over their partner.”	
**Risk factors linked to family violence during COVID-19**					
	Drug abuse	overdose domestic, drug abuse, violence drug, addiction domestic		“@CaesarPodcast As I've noted for years, the history of domestic violence and drug abuse was enough to make him a prime suspect.”	
	Alcohol abuse	liquor shops, violence alcoholism, suicide alcohol, violence alcohol		“March 2020 saw a surge in 'reported' cases of domestic violence. Alcoholism increases chances of abuse manifold on women’s and children.”	
	Financial constrain	violence unemployment, financial ruin, job lost, violence financial		“Take measures to stop domestic violence, which is on the rise due to lock down pressures. No work and no income. Women are facing double burden of providing food for the family and also facing the violence created by the frustrations.”	
	Guns	gun control, violence guns, gun laws, gun violence, violence gun		“During this COVID-19 pandemic we are seeing a rise in gun sales, and a drastic increase in domestic violence cases nationwide.”	
	Trafficking	human trafficking, sex trafficking, trafficking domestic		“This is part of our anti-trafficking #COVID19 response and our new DV response and led by CM @AbbieKamin, @hawctalk.”	
	COVID-19 related	people stuck, violence covid19, unsafe home, abuse quarantine		“During the lockdown domestic violence happens, because the stress and also by the fact that coworkers/friends won’t see the bruises.”	
**Victims of family violence during COVID-19**					
	LGBTQ^c^	trans people, trans women, men men, lesbian couples		“COVID-19 has serious consequences for cis and trans women everywhere including higher risks a result of …the rise in domestic violence.”	
	Women and women of color	women disproportionately affected, beat wife, black women, female victims, women die		“COVID-19 induced isolation and quarantine disproportionately affect women and girls. Around the world, there has been an increase in sexual and gender-based violence during COVID-19.”	
	Refugee women	refuge domestic, charity refuge, violence refuges		“Refugee women are at greater risk for gender-based violence during the COVID-19 lockdown #WorldRefugeeDay https://t.co/iUPeae1vo8”	
	Children	violence child, child abusers, abuse child		“Evidence shows that violence against children is increasing due to #COVID19 lockdown. The pandemic shouldn't create another pandemic of torture and Rights abuse against children.”	
**Social services for victims of family violence during COVID-19**					
	Hotline numbers called	877 863, 863, 6338, 1 800, 800 799, 799 7233, 799 safe, hotline 800, 800 273, 273 8255		“Domestic violence help is available during #COVID19. Call the @ndvh hotline at 1-800-799-7233, text LOVEIS to 22522, or log on to chat at https://t.co/KhIhLk0fq3. You are not alone. https://t.co/qKRP8i3wbr.”	
	Resources	social workers, social service, safety plan, crisis center, limited access, service open		“Risk of abuse through coercive control’s, addictions’; mental health. No social work input is disappointing https://t.co/SbRhrpRlIE.”	
	Shelters	women shelter, violence shelters, seeking shelters, shelters open		“Shelters have formed a GBV safety plan as victims of domestic violence are likely to be forced to stay at home with an abuser for longer periods due to the lockdown. https://t.co/wUQPsKeC4L.”	
	Funding	funding support, consider donating, raise funds		“…The beauty industry supports victims of dv during the COVID-19 by donating products to women's shelters and DV service organizations.”	
	Social media	visit website, violence website, retweet help		“…National Domestic Violence Helpline is there for victims, but it's not for men. That is apparent from their website. https://t.co/bhYUbfOZmv”	
	First responders: social workers	social workers, responding domestic, violence call		“Social workers would just get shot along with the wife or family, so who should society send to domestic violence calls? Cops say domestic violence calls are the most dangerous”	
**Law enforcement responses**					
	Law enforcement	contact police, protection orders, 911 calls, police arrest, legal aid, protective orders, local police		“Calls to local police departments are up in the last month. Help is available. Visit: https://t.co/naSNuaDeP3 for a list of local resources. https://t.co/C1KeCOxKGh.”	
	Reports of DV cases	cases reported, abuse reports, report abuse, increase reports		“Due to the #COVID19 lockdown, there are increasing reports that girls and young women are facing gender-based violence…https: //bit.ly/3cgkfDt.”	
**Social movements and awareness**					
	Support victims	help victims, support victims, campaign combat, zero tolerance, care victims, ask help, reach out, situation help, protect vulnerable		“COVID-19 Lockdown witnesses a global rise in Domestic Violence. Trapped at home with abusers at all hours, lacking privacy to reach out for help, and the sudden disappearance of regular support systems, has isolated individuals, specifically women and children, in violence. https://t.co/80Az37rMvy.”	
	Awareness	awareness domestic, raise awareness, help raise, awareness month, raising awareness, spread awareness, assault awareness		“Women share horrific photos of injuries to raise awareness of domestic violence as 14 are killed since start of lockdown. pics of their horrendous injuries to raise awareness of dv, as killings in the home DOUBLED during the first three weeks of lockdown in the UK. https://t.co/8nOczKoWiu.”	
**Domestic violence–related news**					
	Personnel and events	Johnny Depp, Tracy McCarter, Rikers Island, Melissa DeRosa, Keith Ellison, Chris Brown, Antonio Guterres, Alexandra McCabe, Robert Goforth, Tara Reade, Breann Leath, California’s bail rules		“Today, Secretary to the Governor Melissa DeRosa issued a report to Governor Cuomo outlining the COVID-19 Domestic Violence Task Force's initial recommendations to reimagine New York's approach to services for domestic violence survivors. https://t.co/VpMJEd7Njc.”	

^a^DV: domestic violence.

^b^FGM: female genital mutilation.

^c^LGBTQ: lesbian, gay, bisexual, transgender, and queer or questioning.

### Increased Vulnerability: COVID-19 and Family Violence

Tweets mentioning rising rates of domestic violence as a consequence of COVID-19 were frequent, with popular bigrams like “violence increased,” “violence higher,” “rising violence,” and “violence skyrocketing.” Increases in hotline calls and reports of family violence were also influenced by the ongoing COVID-19 pandemic (eg, calls increased, calls help, reports surge). A representative tweet indicated, “a Miami Valley nonprofit agent is seeing a spike in crisis line calls during the pandemic.” Other consequences of the pandemic include homicides related to domestic violence and mental health issues (eg, depression, mental abuse).

### Types of Family Violence During COVID-19

Findings showed that several types of family violence were mentioned together in a single tweet alongside terms related to COVID-19, such as “child abuse/maltreatment” (eg, assault child, rape child), “domestic violence” (eg, abusive partners, violence partners), and “sexual violence” (eg, sexually assault, marital rape).

### Forms of Family Violence During COVID-19

Two primary forms of family violence were discussed on Twitter during the COVID-19: “physical aggression” (eg, physically hurt, stop hitting) and “coercive control” (eg, power control, forced stay). The latter is demonstrated by this example: “…abusers may use more coercive control tactics surrounding the #covid19 pandemic to continue to maintain power and control over their partner.”

### Risk Factors Linked to Family Violence During COVID-19

We found that the rising rate of domestic violence was associated with risk factors: “drug abuse,” “alcohol abuse,” “financial constraints” (eg, job loss, loss income), “guns,” “trafficking,” and “COVID-19 related” (eg, lockdown, stuck home, quarantine). Sample tweets include “March 2020 saw a surge in reported cases of domestic violence. Alcoholism increases chances of abuse manifold on women and children…” and “During the lockdown, domestic violence happens because the coworkers/friends can’t see the bruises.”

### Victims of Family Violence During COVID-19

Tweets designated the LGBTQ (lesbian, gay, bisexual, transgender, and queer or questioning) community, women, women of color, refugee women, and children as victims of family violence during the COVID-19. Popular words in describing the victims and survivors of family violence included “trans people,” “lesbian couples,” “women disproportionately affected,” “beat wife,” “black women,” “female victims,” “refuge domestic,” “charity refuge,” “violence child,” “child abusers,” and “abuse child.”

### Social Services for Victims of Family Violence During COVID-19

Social services for victims of family violence was a prominent theme discussed by Twitter users during the pandemic, as indicated by the high frequency mentions of hotline numbers. Resources, shelters, funding support, and visiting websites on family violence were also frequently mentioned in tweets. In addition, confidential services, safety plans, and limited access were representative topics identified in the sampled tweets. Social workers’ safety was tweeted as a salient topic in our data set: “…domestic violence cases are just asking for a lot of social workers to get shot and killed” and “Has anyone actually asked social workers how willing they are to go on domestic violence calls…?”

### Law Enforcement Responses

With the rising rates of family violence during the pandemic, reports of domestic violence cases (eg, cases reported, abuse reports, violence reports, increase reports, and reported increase) were a salient topic in the tweets. Police departments (eg, police officers, local police, police chief, 911 calls, contact police, police arrest) were the first responders on the front lines during increased domestic violence reports during COVID-19.

### Social Movements and Awareness

Findings also identified social justice movements and awareness to support victims and survivors of family violence. Tweet content highlighted the advocacy of zero tolerance for domestic violence, indicated by popular bigrams such as “help victims,” “campaign combat,” “violence advocacy,” “care victims,” “raise awareness,” and “awareness campaign” and sample tweets like “Women share horrific photos of injuries to raise awareness of domestic violence.”

### Domestic Violence–Related News

News events related to domestic violence cases during the pandemic were also identified, such as (1) American actor Johnny Depp’s denial of domestic abuse allegations by ex-wife Amber Heard; (2) Tracy McCarter’s murder charge for the fatal stabbing of her husband in Manhattan; (3) singer Chris Brown’s arrest in Paris on allegations of rape; (4) Tara Reade’s sexual assault allegations against Joe Biden; (5) Kentucky legislator Robert Goforth’s arrest for 4th-degree domestic violence; and (6) death of police officer Breann Leath, who was shot on duty while responding to a domestic disturbance call.

News of solutions to help survivors of domestic violence were also frequently discussed in the sampled tweets. For example, the governor of the New York State Council on Women and Girls, Melissa DeRosa, created a task force to find innovative solutions to the domestic violence spike during the COVID-19 pandemic. United Nations chief Antonio Guterres called for measures to address the surge in domestic violence linked to lockdowns that were imposed by governments in responding to the COVID-19 pandemic.

The news article *Child abusers eligible for immediate release under California’s new $0 cash bail emergency mandate* [47] has become a prominent topic due to the high volume of retweets. Given the new state rules, individuals arrested for child abuse will be released on $0 bail in California. The original tweet was posted by Bill Melugin (@billFOXLA) and had been retweeted almost 1000 times (“RT @BillFOXLA: Under California's new $0 cash bail rules, child abusers are now eligible for immediate release. San Bernardino County Sheriff @sheriffmcmahon tells me he had to release a felony child abuse suspect /w priors for domestic violence & child abuse immediately after arrest. @FOXLA”).

## Discussion

### Principal Results

Our study employed a large-scale analysis of tweets on public discourse related to family violence on Twitter during the COVID-19 pandemic. The study's Twitter data consisted of a random selection of more than 1 million tweets mentioning family violence and COVID-19 from April 12 to July 16, 2020. The machine learning technique LDA was used to extract a high volume of co-occurring word pairs and topics related to family violence from unstructured tweets. The study contributes to the understanding of public discourse and concerns of family violence during the COVID-19 pandemic. We identified 9 themes from the analysis: (1) increased vulnerability: COVID-19 and family violence (eg, increasing rates, victims affected); (2) types of family violence; (3) forms of family violence; (4) victims of family violence; (5) risk factors linked to family violence; (6) social services for victims of family violence; (7) law enforcement responses; (8) social movements and awareness; and (9) domestic violence–related news. The study adds to existing scholarship, where there is a lack of data on the COVID-19–domestic violence connection, or only anecdotal reports. Our findings contribute to understanding family violence during the pandemic by providing surveillance via tweets, which is essential to identify potentially effective policy programs in offering targeted support for victims and survivors and preparing for future outbreaks.

Twitter users have discussed who is at higher risk of family violence during the lockdown. Findings reveal a broader range of affected victims, such as the LGBTQ community. Salient tweets suggest that women and children are disproportionately affected by family violence that is consistent with the majority of the research in the field [38,48-51]. Violence against children has been associated with previous epidemics [6]. In addition, the sampled tweets suggest that domestic violence–related discussions focus on the support and protection of victims instead of interventions against abusers, consistent with one recent study using Twitter data for domestic violence research [39]. We find tweets mentioning family violence and COVID-19 have a limitation in primarily posting stories about male-to-female violence [37] even though other patterns of violence exist, including female-to-male, male-to-male, and bidirectional IPV [52].

Tweets about family violence and COVID-19 during the lockdown mentioned a range of risk factors associated with family violence during pandemics, such as drug abuse, alcohol abuse, financial constraints, guns, and trafficking. Our study reveals similar results with one recent report by Peterman and colleagues [10], who summarized that 9 main pathways that connect the COVID-19 pandemic and violence against women and children (ie, economic insecurity and poverty-related stress; quarantines and social isolation; disaster- and conflict-related unrest and instability; and inability to temporarily take shelter from abusive partners). For example, public discussions indicate that alcohol abuse continues to be a risk factor for family violence during stressful events [53]. Financial constraints (eg, financial ruin, lost jobs, economic collapse) due to COVID-19 create barriers for victims of family violence for help seeking [2]. Beland and colleagues [54] analyzed the Canadian Perspective Survey Series and found that financial worries due to COVID-19 contributed to increased family violence and stress. An increasing rate of domestic homicides identified in tweets suggests that guns are still a concern at home where family violence occurs. Specific COVID-19–related risk factors (eg, quarantines, social isolation) limit contact between victims of family violence and the outside world, trapping them at home with their abusers; these factors were indicated by the frequent use of words like “people stuck,” “unsafe home,” “people locked,” and “abuse quarantine” on Twitter.

Multiagency integration of law enforcement responses (eg, protection orders, arrest), social services (eg, hotlines, shelters), and social movements and awareness are recommended to address domestic violence and support victims [55]. Social services (including deployment of social work practitioners, therapists, etc) for cases of domestic violence must be resourced during the pandemic. Due to the mobility restriction, a lack of informal support, such as that from family, friends, coworkers, further contributes to increased rates of family violence during the pandemic. Thus, it is more crucial than ever for victims to access voluntary sector practitioners' support during the COVID-19 pandemic [8]. Our results provide evidence that some agencies continued to deliver services during the pandemic. For example, several hotline numbers in the United States have been frequently mentioned during the pandemic, such as “Illinois Domestic Violence Hotline, 877-863-6338 (877-TO END DV),” “National Suicide Prevention Lifeline, 800-273-8255 (US),” “National Domestic Violence Hotline, 800-799-SAFE (7233) (US),” “National Sexual Assault Telephone Hotline, 800-656-HOPE (4673) (US),” and “Loveisrespect, Text LOVEIS to 22522 (US).” We also identified popular hotline numbers from the United Kingdom, such as Mind the Mental Health Charity (Mind Infoline: 0300-123-3393), the National Stalking Helpline (0808-802-0300), and the National Domestic Abuse Helpline (0808-2000-247). However, a commentary in the *Canadian Medical Association Journal* raises concerns about family violence support using videoconference or telemedicine settings where the abusers can be present [56]. Abusers can coercively control victims-survivors’ use of mobile phones to access hotline support. Therefore, further evidence is needed to indicate whether the services fulfill their roles.

Twitter conversations about highly publicized domestic violence cases were significant. News about Hollywood star Johnny Depp’s denial of abuse allegations when he was accused of domestic violence against his ex-wife Amber Heard was a prominent topic in the sampled tweets. Our results show public discussions of high-profile cases of domestic violence (eg, athletes arrested for domestic violence), consistent with previous studies. Cravens et al [37] used qualitative content analysis to examine the factors that influence IPV victims to leave an abusive relationship using 676 tweets related to #whyIstayed and #whyIleft. Xue et al [38] analyzed 322,863 tweets about domestic violence and found that high-profile cases such as Greg Hardy's domestic violence case are prominent. These studies consistently show that Twitter continues to be a source of news coverage on current events for domestic violence, even during the COVID-19 pandemic.

### Limitations

There are a number of limitations to this study that must be acknowledged. First, Twitter data reveal insights from Twitter users and thus does not represent the entire population's opinions. Despite this shortcoming, our study provides one of the first large-scale analysis of tweets using real-time data to identify the impact of COVID-19 on family violence. Second, we did not include non-English tweets in the analysis. Future studies should carry out analyses on non-English tweets regarding the impact of COVID-19 on family violence. Third, even though our collected data cover 90 days of the outbreak since April 12, 2020, discussion patterns may evolve as the COVID-19 situation continues to change over time. Fourth, the search terms used in the study mostly reflect terminology used by professionals rather than victims when discussing family violence. For example, one study examined how child abuse victims post their stories on social media and found that the victims rarely use explicit words to describe their experiences [57]. Thus, this study may be limited in capturing victims’ opinions. To protect Twitter users' privacy and anonymity, we did not examine the sample's sociodemographic characteristics. It remains unknown whether the collected tweets were from victims, abusers, organizations, etc. It is also possible that abusers may prevent victims from reaching out for help on social media [9]. Future studies could consider sampling tweets from victims of family violence to further examine the impact of COVID-19.

### Conclusion

As seen in our large-scale tweets data set, people have been actively discussing family violence in the context of COVID-19. We identified 9 themes and 33 topics relating to family violence and COVID-19. The findings demonstrate that Twitter can serve as a platform for real-time and large-scale surveillance of family violence by offering an understanding of the people who are discussing the impact and risk factors associated with COVID-19, which is essential for developing policy programs for supporting victims and survivors. This study provides insights for professionals who work with victims and survivors of family violence to develop a social network–based support system for informal and formal help when conventional in-person support services become unavailable during future outbreaks.

## Appendix

Multimedia Appendix 1 Hashtags used as data collection search terms.

Multimedia Appendix 2 Notations for Latent Dirichlet Allocation (LDA).

## Abbreviations

LDA: Latent Dirichlet Allocation
LGBTQ: lesbian, gay, bisexual, transgender, and queer or questioning
