# Supplementary material for: The Hidden Pandemic of Family Violence During COVID-19: Unsupervised Learning of Tweets
Source: J Med Internet Res. 2020 Nov 6;22(11):e24361. doi: 10.2196/24361 (PMC7652592; doi:10.2196/24361)
Supplement: Multimedia Appendix 1 [file jmir_v22i11e24361_app1.docx]

**Appendix 1 Hashtags used as data collection search terms**

| **Hashtag** | **Count** |
| --- | --- |
| #COVID19 | 29,209,496 |
| #Coronavirus | 11,226,976 |
| #lockdown | 2,320,553 |
| #StayHome | 1,851,872 |
| #Quarantine | 873,250 |
| #StaySafe | 642,118 |
| #COVID | 573,685 |
| #Covid_19 | 568,208 |
| #pandemic | 390,184 |
| #StayAtHome | 267,038 |
