# Supplementary material for: The Hidden Pandemic of Family Violence During COVID-19: Unsupervised Learning of Tweets
Source: J Med Internet Res. 2020 Nov 6;22(11):e24361. doi: 10.2196/24361 (PMC7652592; doi:10.2196/24361)
Supplement: Multimedia Appendix 2 [file jmir_v22i11e24361_app2.docx]

**Appendix 2. Notations for Latent Dirichlet Allocation (LDA)**

| Notation | Meaning |
| --- | --- |
| $M$ | Number of documents. |
| $N_{i}$ | Number of linguistic units in the i-th document. |
| K | Number of topics. |
| $\sim Dir\left( \cdot\right)$ | Sampling from a Dirichlet distribution. |
| $\sim\mathrm{Multinomial}\left( \cdot\right)$ | Sampling from a multinomial distribution. |
| $\theta_{i}$ | Distribution of topic for the i-th document. |
| $\phi_{i}$ | Distribution of words in the k-th topic |
| $\alpha,\beta$ | Parameters for the Dirichlet distributions. |
| $z_{i,j}$ | The topic for the j-th linguistic unit in the i-th document. |
| $w_{i,j}$ | The the j-th linguistic unit in the i-th document. |
